# Protective effects of early low-intensity exercise on the antigravity soleus muscle following focal cerebral infarction in rats

**DOI:** 10.3389/fresc.2026.1789922

**Published:** 2026-07-29

**Authors:** Fumiyo Matsuda

**Affiliations:** Department of Physical Therapy, School of Health Sciences, Faculty of Medicine, Kagoshima University, Sakuragaoka, Kagoshima, Japan

**Keywords:** muscle fiber type, rehabilitation, skeletal muscle, soleus, stroke

## Abstract

**Background:**

Skeletal muscle changes following stroke are a major factor influencing subsequent motor recovery.

**Objective:**

To examine the effects of early low-intensity exercise on motor function, neurological deficits, soleus muscle mass, fiber-type composition, and muscle fiber cross-sectional area following experimental stroke.

**Methods:**

Adult male Wistar rats were randomly assigned to ischemia-control (IC; *n* = 23) and ischemia-exercise (IE; *n* = 23) groups. Stroke was induced 90 min after occlusion of the left middle cerebral artery. The next day, the rats ran on a treadmill for 20 min once a day for up to 28 days. The beam-walking test and a neurological evaluation scale were used to assess post-ischemia functional recovery. The muscle fiber characteristics of the soleus of both sides were examined using histochemical and morphometric methods at 1, 3, 5, 7, 14, and 28 days post-stroke.

**Results:**

Compared to that in the IC group, the motor recovery and neurological function on day 28 were significantly greater in the IE group. The infarct volumes were initially comparable; however, the IE group exhibited a significant reduction beginning on day 5, and two-way ANOVA confirmed smaller infarct volumes in the IE group overall. The soleus muscle weight, fiber-type distribution, and cross-sectional area showed no significant temporal or between-group differences.

**Conclusions:**

Early exercise intervention promotes functional recovery and may attenuate lesion progression as assessed by TTC staining without detectable deterioration of soleus muscle morphology, highlighting the potential value of safe early mobilization after stroke.

## Introduction

Stroke remains a leading cause of long-term disability worldwide, and although advances in acute medical care have reduced mortality, a growing number of survivors experience persistent motor impairment and reduced quality of life (1) and patients in many countries suffer from permanent motor paralysis and incur huge costs for long-term treatment and rehabilitation (2). Skeletal muscle alterations following stroke, including muscle atrophy and fiber-type shifts, are commonly observed and are known to contribute to long-term functional decline and compromised rehabilitation outcomes. These peripheral changes are traditionally considered a major determinant of post-stroke motor recovery.

Clinical data strongly support early mobilization and training, and appropriate rehabilitative interventions can lead to some degree of improvement (3, 4). Several studies have demonstrated the beneficial effects of early exercise on ischemia-induced brain injury in animal models (5–7). Following an acute stroke, skeletal muscle atrophy is associated with prolonged disability and reduced functional ability in patients (8, 9). Evaluating the brain and changes in the skeletal muscle fiber types involved in functional recovery after stroke is important for selecting the best rehabilitation strategy. However, the mechanisms underlying histochemical changes in muscle fibers according to functional demands are poorly understood. Moreover, the optimal timing, intensity, and physiological targets of early exercise interventions remain controversial, particularly in the acute-to-subacute phases following stroke. Current research on skeletal muscle atrophy following cerebral infarction is limited, as animal studies primarily focus on elucidating the molecular biological mechanisms (10), with little research conducted on temporal changes in skeletal muscle. Human studies have investigated skeletal muscle atrophy following cerebral infarction sequelae over time, revealing that, in the long term (3), individual factors play a strong role, with the involvement of multiple complex factors such as pre-onset lifestyle habits. Consequently, various viewpoints are commonly present (10–12).

In parallel, stroke-induced skeletal muscle alterations have attracted growing research attention. Muscle atrophy is more pronounced in the skeletal muscles on the paralyzed side after stroke onset and can occur within weeks to months (3). Changes in muscle fiber type [major histocompatibility complex (MHC)-type isoforms of muscle fibers] (3) occur because of reduced activity after stroke (13) and alterations in innervations (14). Changes in skeletal muscle following stroke are a major factor influencing subsequent motor recovery in patients with stroke (15), which complicates stroke rehabilitation. Patients with chronic stroke exhibit reduced overall cross-sectional area (CSA) in paralyzed skeletal muscles, along with decreased content of Type I and Type IIA fibers and a shift toward Type IIX fibers (16). Physical exercise may ameliorate neurological impairment by impeding neuronal loss following various brain insults, and has now been proposed as a possible treatment option for such conditions, especially in patients with stroke. Exercise has beneficial effects on brain function, including promoting plasticity. Several experimental studies have demonstrated that appropriately prescribed exercise promotes neuroplasticity, reduces secondary degeneration, and enhances post-stroke recovery (17–21). Previous studies have reported muscle atrophy, reduced cross-sectional area, and fiber-type transitions toward fast-twitch phenotypes in paretic muscles of stroke survivors. Nevertheless, most animal studies have focused on molecular mechanisms underlying muscle atrophy, while relatively few studies have examined temporal changes in skeletal muscle morphology in relation to functional recovery, especially during the early post-stroke period. Moreover, human studies are often confounded by heterogeneous pre-stroke activity levels, comorbidities, and rehabilitation regimens, limiting mechanistic interpretation.

Importantly, the relationship between central nervous system recovery and peripheral skeletal muscle adaptation after stroke remains incompletely understood. While functional recovery is often implicitly assumed to reflect both neural and muscular improvements, it is unclear whether early exercise-induced neurological benefits necessarily coincide with structural adaptations in skeletal muscle. Emerging evidence suggests that central and peripheral responses to exercise may follow different time courses and may be driven by distinct mechanisms.

Therefore, the present study aimed to investigate the effects of very early, low-intensity treadmill exercise initiated 24 h after transient middle cerebral artery occlusion in rats, focusing on both central outcomes (neurological function and infarct volume) and peripheral outcomes (soleus muscle weight, fiber-type distribution, and fiber cross-sectional area) over a 28-day period. We hypothesized that early exercise would improve neurological recovery and attenuate infarct progression, while skeletal muscle (soleus) structural adaptations might be minimal under low-intensity conditions during the early recovery phase. The findings of this study could provide insight into developing approaches to advance the care and treatment of stroke.

## Methods

### Animals

This study used 46 male Wistar rats (Japan SLC Inc., Tokyo, Japan) weighing 220–260 g (approximately 8 weeks old) at the time of surgery. The rats were randomly assigned to ischemia-non-exercised control (IC) (*n* = 23; *n* = 3 at 1, 3, 5, 7, and 14-day time points; *n* = 8 at 28 days) and ischemia-exercise (IE) (*n* = 23; *n* = 3 at 1, 3, 5, 7, and 14-day time points; *n* = 8 at 28 days) groups before surgery. The IC and IE rats had left MCA occlusion, and the IE rats were placed on a treadmill. None of the rats died due to treadmill exercise or disease between 24 h after the stroke and at the end of the experiments. The animals were socially housed (2–3 rats per cage) under a 12-h reverse light/dark cycle in clear Plexiglas cages with food and water available *ad libitum*. Experimental protocols were performed in accordance with the guidelines established by the Animal Care Committee of the Ethics Board of the Institute of Laboratory Animal Sciences of Kagoshima University.

The ischemic stroke model procedures were performed according to the methods described previously (22, 23).

The animals were anesthetized via the injection of 4% chloral hydrate (10 mL/kg, intraperitoneally) and were administered further doses as necessary to ensure adequate anesthesia during surgery. Rectal temperature was monitored throughout the surgical procedure and maintained at 37 °C using a heating blanket controlled by an electronic temperature controller (KN-474, NATUME, Tokyo, Japan). Stroke was induced by left MCA occlusion for 90 min using an intraluminal filament. Briefly, a midline incision was made to expose the left common carotid artery (CCA), external carotid artery (ECA), and internal carotid artery (ICA). The distal ECA branch was completely coagulated. The CCA and ECA were tied using white threads. A monofilament (4-0 nylon suture with a blunted tip of 0.2–0.3 mm diameter and 16-mm length) was inserted into the left CCA via arteriotomy. It then passed through the ICA into the intracranial circulation and lodged in the narrow proximal segment of the anterior cerebral artery (ACA), blocking the MCA at its origin. After 90 min of MCA occlusion, reperfusion was established by withdrawal of the filament. Regional cerebral blood flow (rCBF) was measured using a laser Doppler flowmeter (ALF-21, Advance Co. Inc. Tokyo, Japan) before and during MCAO. A probe in the shape of a flat rectangular sheet (7.5 mm, 3.5 mm, and 1.0 mm in length, width, and thickness, respectively) was placed over the ischemic side in the natural pocket formed between the temporal muscle and the lateral aspect of the skull. All animals exhibited a significant reduction in blood flow (greater than 20% reduction compared to the pre-ischemic baseline) during MCA occlusion, and none were excluded from the study because of a lack of reduction in blood flow.

### Training

Rats in the IC group remained in their home cages without exercise. Rats in the IE group began treadmill running 24 h after surgery and exercised for 20 min/day for 28 days. A motorized treadmill (RR-1200, AKK, Japan) was used, and the running speed was progressively increased (3 m/min on days 1–3, 8 m/min on days 4–6, and 13 m/min thereafter; 0° incline). Animals were housed two per cage under standard conditions with free access to food and water. The body weight was monitored periodically (24), and no animals were excluded due to an unwillingness to run.

### Motor behavior and neurological assessments

Animals from the IE and IC groups were examined using a motor test paradigm (limb placing: motor behavior test), and neurological deficits were assessed before and 1, 3, 5, 7, 14, and 28 days after surgery. All rats were tested thrice on each trial day.

Motor behavior was assessed using a beam-walking task based on the scoring system of Fenny et al. (25), with minor modifications (23). All animals were trained before surgery to ensure normal baseline performance.

A neurological grading system with a five-point scale (0–4) described by Menzies et al. (23, 26) was used: 0 = no apparent deficits; 1 = right forelimb flexion; 2 = decreased grip of the right forelimb while tail pulled; 3 = spontaneous movement in all directions while right circling only if pulled by the tail; and 4 = spontaneous right circling.

Of the two observers who rated motor behavior and performed neurological assessments, one was unaware of which rats belonged to the IC and IE groups, and her scores were mainly used for subsequent analyses.

### Infarct assessment

The rats in the IC and IE groups were randomly sacrificed at 1, 3, 5, 7, 14, and 28 days (*n* = 3 at each time point at 1, 3, 5, 7, and 14 days; *n* = 8 at 28 days) after ischemia induction. At each time point, rats were euthanized under deep anesthesia by an intraperitoneal overdose of pentobarbital sodium (150 mg/kg), in accordance with the AVMA Guidelines for the Euthanasia of Animals (2020) and institutional animal care regulations. The infarct volume was quantified using a 2% solution of 2,3,5-triphenyltetrazolium chloride (TTC) staining according to established methods (23, 27), and an indirect calculation method was used to correct for edema.

### Sample preparation

The soleus muscles in both legs (left and right) were dissected, cleaned of fat and connective tissue, weighed, quickly frozen in isopentane, and chilled with liquid nitrogen. The muscle was then stored at –80 °C until analysis, and the muscle from both sides was analyzed for all animals.

### Histological analysis

The soleus muscle was vertically mounted on cork plates in tragacanth gum jelly of appropriate softness (28) to obtain cross-sections. Transverse serial sections (10-μm thick) were cut with a cryostat microtome at –20 °C and stained with hematoxylin and eosin for general observation. The sections were also stained for myosin adenosine triphosphatase (ATPase, pH 10.6) according to the method of Guth and Samaha (29) with some modifications, and the muscle fibers were classified as Type 1 or 2 fibers.

Transverse sections of the soleus muscle were used for the morphometric analyses. The entire cross-section of each soleus muscle stained with ATPase was photographed at 20× magnification to determine the fiber-type composition. The central region of the soleus muscle was photographed at 50× magnification, and all muscle fibers delineated by the entire fiber boundary were measured for the cross-sectional area. A random sample of 100–120 fibers from each soleus muscle was analyzed.

The number (%) of fibers with photological, morphological, and histochemical alterations was determined by hematoxylin and eosin staining and ATPase preparation.

The fiber cross-sectional area was determined using a computer-assisted image analyzer with ImageJ (version 1.53a; NIH, USA). The number of fibers was counted using a computer-assisted image analyzer with ADOBE® PHOTOSHOP® 5.0 (Adobe Systems Inc., San Jose, CA, USA). All the histological analyses were performed in a blinded manner.

### Statistical analysis

Motor behavior and neurological assessment scores are ordinal variables and are therefore presented as medians (ranges).

Although non-parametric methods are generally recommended for ordinal outcomes, the present study aimed to evaluate both between-group differences and temporal changes simultaneously. Therefore, a two-way ANOVA followed by Dunnett's *post hoc* test was applied to provide a consistent framework for assessing group and time effects.

Given the ordinal nature of these variables and the limited sample size at several early time points (*n* = 3), these results should be interpreted with caution and regarded as exploratory.

Continuous variables are presented as mean ± standard deviation and were analyzed using two-way ANOVA followed by Dunnett's *post hoc* test when appropriate. Statistical analyses were performed using IBM SPSS Statistics for Windows, version 29.0 (IBM Corp., Armonk, NY, USA).　Statistical significance was set at *p* < 0.05.

## Results

### Change in body weight

The pre-stroke body weights were 253.61 ± 32.30 g in the IC group and 246.22 ± 14.60 g in the IE group, with no significant difference (*p* = 0.33). The mean percentage weight loss after cerebral infarction (1–28 days) was 97.1% ± 31.8% in the IC group and 94.9% ± 28.2% in the IE group, with no significant difference (*p* = 0.81). The postoperative weight reduction rate peaked at 7 days postoperatively in both groups, after which it gradually returned to the preoperative weight. Specifically, 28 days post-surgery, the IC group showed a significant weight gain of 130.3% ± 15.5% (*p* = 0.001), and the IE group showed a significant weight gain of 127.8% ± 15.7% (*p* = 0.001), compared to pre-surgery levels. However, no significant difference in weight gain rate was observed between the two groups (weight ratio, *p* = 0.77). These results indicate that while cerebral infarction caused temporary weight loss, weight increased significantly beyond preoperative levels by 28 days postoperatively, regardless of exercise status (Data not shown). These individuals were able to perform daily activities, including feeding and elimination, independently and without difficulty, and were free from stress due to the effects of surgery, feeding difficulties caused by motor paralysis, or stress from motor interventions.

### Motor behavior and neurological assessments

The time-dependent results of the motor behavior are presented in Table 1A. One day after surgery, motor paralysis occurred in both groups, with all patients scoring 0. Subsequently, gradual recovery from motor paralysis was observed; in the IE group, all animals achieved a score of 1 after 7 days. Both groups showed recovery of motor behavior over time. On day 28, the IC group had a score of 0–1, whereas the IE group had a score of 1–4, indicating significantly greater recovery of motor behavior in the IC group (*p* = 0.0079).

**Table 1:** Motor and neurological scores after middle cerebral artery occlusion in rats.

(A)	Ischemia-exercise (IE)	Ischemia-non-exercised control (IC)
Pre-operation	5 (5)	5 (5)
1 day	0 (0)	0 (0)
3 days	0 (0–1)	0 (0–1)
5 days	0 (0–1)	0 (0–1)
7 days	1 (1–2)	1 (0–1)
14 days	2 (1–2)	1 (0–1)
28 days	1.5 (1–4)	1 (0–1)
(B)	IE	IC
Pre-operation	0 (0)	0 (0)
1 day	2 (2–3)	2 (2–3)
3 days	2 (1–2)	2 (1–2)
5 days	1 (1–2)	2 (1–2)
7 days	1 (0–1)	1 (0–2)
14 days	0 (0–1)	1 (0–2)
28 days	0 (0–1)	1 (0–2)

Motor (A) and neurological (B) scores are expressed as medians (range). Motor function declined after ischemia but improved over time (A), and neurological deficits increased initially and gradually returned toward baseline (B). Statistical analysis revealed significant differences between the ischemic exercise (IE) and ischemic non-exercised control (IC) groups at 28 days (*p* < 0.05) (A and B). The sample sizes were *n* = 23 preoperatively, *n* = 3 at each postoperative time point (days 1, 3, 5, 7, and 14), and *n* = 8 at day 28 for both groups.

The time-dependent results of neurological assessments are presented in Table 1B. One day post-surgery, both the IC and IE groups showed severe neurological deficits, with scores of 2–3. Subsequently, gradual recovery of neurological findings was observed, which was significantly greater in the IE group than in the IC group at 28 days (*p* = 0.0073). Specifically, the IC group reached a plateau after 7 days, whereas the IE group continued to show neurological recovery beyond 7 days. Intra-group comparisons at 1–28 days post-surgery revealed significant postoperative recovery in the IE group (*p* < 0.001) compared to the IC group (*p* = 0.17).

### Infarct volume

**Figure 1:**
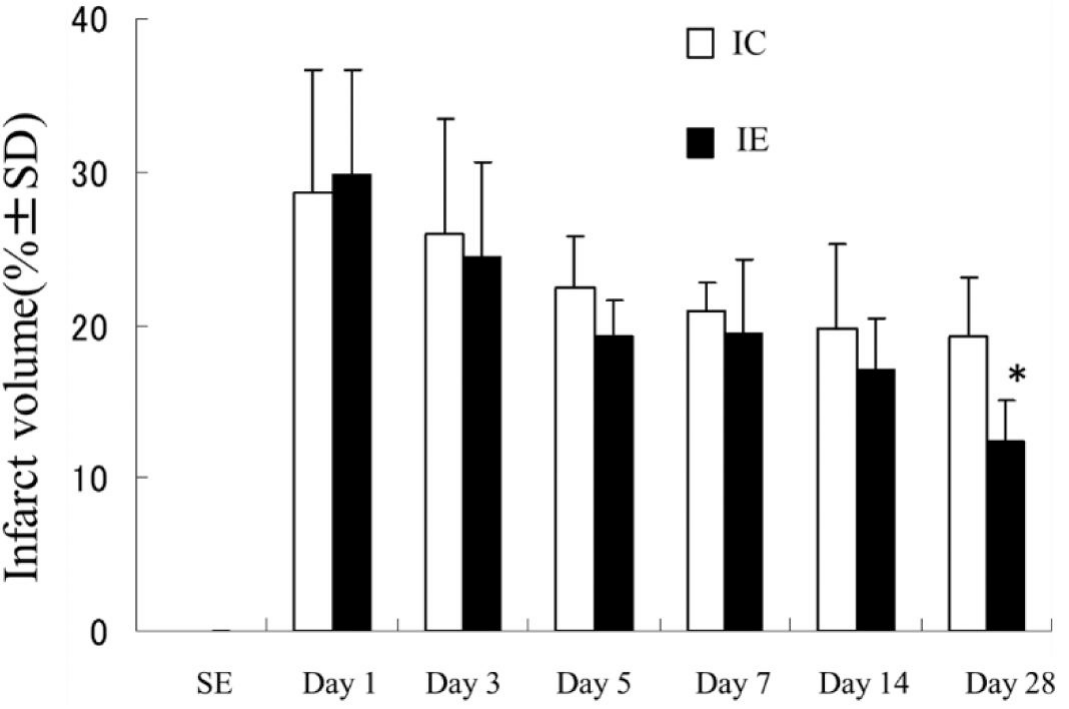
Cerebral infarction volume (%). In the two-way ANOVA, the IE group showed a significantly smaller TTC-defined lesion volumes compared with the IC group (*p* = 0.024). ANOVA; IC, Ischemic non-exercised control'; IE, Ischemic exercise.

Histological analysis with TTC staining was performed to determine the extent of brain infarction at 1, 3, 5, 7, 14, and 28 days after ischemia. The cerebral infarction volume results are shown in Figure 1. The infarct volumes were nearly identical in both groups on postoperative days 1 and 3. Furthermore, cerebral infarction sites were observed in both groups as extensive areas within the left middle cerebral artery territory (from the cortex to the subcortical regions), consistent with those reported in previous studies (25). Subsequently, the IE group showed a significant reduction in TTC-defined lesion volume over time, beginning early (5 days post-stroke) (*p* = 0.0026). However, in the IC group, although the cerebral infarction volume showed a decreasing trend after 14 days, no significant reduction was observed over time (*p* = 0.356). In the two-way ANOVA, the IE group showed significantly smaller TTC-defined lesion volumes compared with the IC group (*p* = 0.024).

### Soleus muscle weight

**Figure 2:**
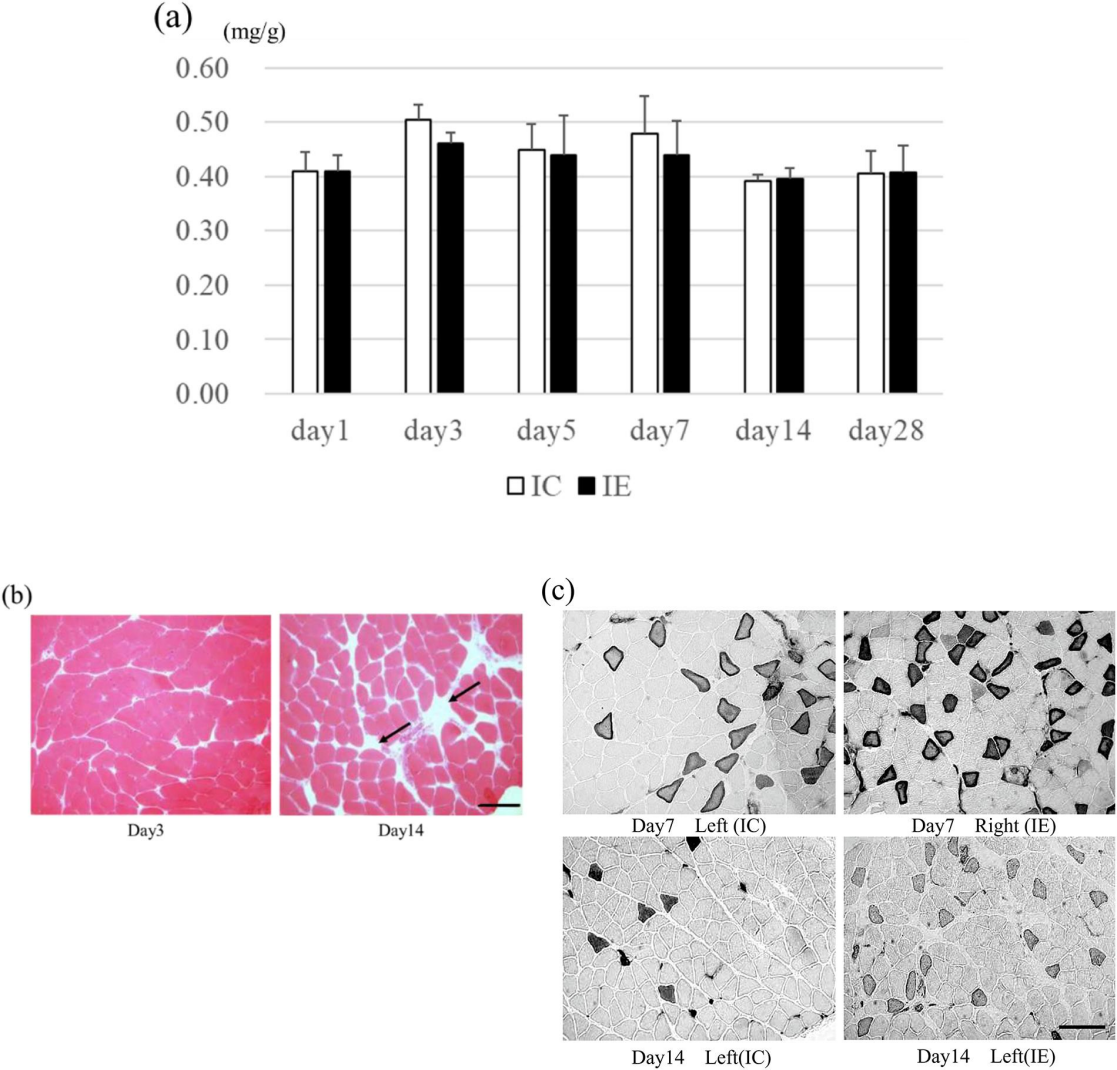
Histological and morphological characteristics of the soleus muscle following cerebral infarction. **(a)** Ratio of soleus muscle mass to body weight. The ratio was calculated by dividing soleus muscle mass by body weight. Although slight fluctuations were observed between days 1 and 28 post-surgery, no significant differences were detected between the ischemic non-exercised control (IC) and ischemic exercise (IE) groups (*p* = 0.85). **(b)** Hematoxylin and eosin (HE) staining of the paralyzed-side soleus muscle in the IC group at 3 and 14 days after cerebral infarction induction. Small gaps between muscle fibers were observed (arrows), but no disruption of overall muscle structure, fiber-size variation, or reduction in cytoplasmic staining intensity was detected. Bar = 300 μm. **(c)** ATPase staining of the paralyzed-side soleus muscle in the IC group at 3 and 14 days post-induction. The proportion of Type II fibers tended to be higher on the paralyzed side than on the contralateral side at 7 and 14 days, although no significant differences were observed. Bar = 300 μm. IC, Ischemic non-exercised control; IE, Ischemic exercise; HE, Hematoxylin and eosin; ATPase, Adenosine triphosphatase.

Figure 2a shows the values obtained by dividing the mass of the soleus muscle by the body weight. The mass of the soleus muscle changes over time, which correlates with fluctuations in body weight. Therefore, the mass of the soleus muscle was calculated by dividing it by the weight. The weight of the soleus muscle tissue divided by body weight showed slight fluctuations on both sides between days 1 and 28 after surgery. However, no specific or significant changes were observed between the sides, between the IC and IE groups, or in temporal comparisons based on postoperative days (*p* = 0.85).

### Soleus fiber-type distribution

No specific or significant changes were observed in the bilateral difference, the comparison between the IC and IE groups, or the temporal comparison based on postoperative days, despite minor fluctuations on both sides from 1 to 28 days postoperatively (*p* = 0.96) (Table 2). HE staining comparing the paralyzed side of the soleus muscle in the IC group at 3 and 14 days post-surgery—the time points showing the greatest difference in muscle weight-to-body weight ratio—revealed gaps between muscle fibers in some muscles at 14 days compared to 3 days post-surgery (Figure 2b). However, no disruption of the basic muscle structure, variation in muscle fiber size, or reduction in cytoplasmic staining was observed. In ATPase staining, Type I fibers accounted for an average of 80%–95% of that observed during the 28-day postoperative period. This showed a tendency toward a lower proportion of Type I fibers than the typical muscle fiber composition of the rat soleus muscle (29). Specifically, at 7- and 14-days post-surgery, the IC group showed a tendency toward a higher proportion of Type II fibers on the paralyzed side than on the contralateral side, although no significant difference was observed (Figure 2c).

**Table 2:** Fiber type distribution (%) of the rat soleus.

Group		Left soleus (unaffected side)		Right soleus (affected side)	
		Fiber-type distribution (%)		Fiber-type distribution (%)	
		Type 1	Type 2	Type 1	Type 2
IE	1 days	86.8 ± 4.9	13.2 ± 4.9	83.4 ± 7.6	16.6 ± 7.6
	3 days	82.0 ± 11.0	18.0 ± 11.0	79.1 ± 7.1	20.9 ± 7.1
	5 days	87.8 ± 4.7	12.2 ± 4.7	83.9 ± 5.9	16.1 ± 5.9
	7 days	87.7 ± 6.9	12.3 ± 6.9	87.9 ± 6.2	12.1 ± 6.2
	14 days	86.0 ± 1.8	14.0 ± 1.8	93.5 ± 0.8	6.5 ± 0.8
	28 days	88.0 ± 9.5	12.0 ± 9.5	84.4 ± 9.8	15.6 ± 9.8
IC	1 days	86.5 ± 8.8	13.5 ± 8.8	86.3 ± 4.7	13.7 ± 4.7
	3 days	90.5 ± 4.1	9.5 ± 4.1	85.1 ± 2.2	14.9 ± 2.2
	5 days	90.5 ± 1.0	9.5 ± 1.0	89.1 ± 1.7	10.9 ± 1.7
	7 days	92.6 ± 7.0	7.4 ± 7.0	82.9 ± 8.3	17.1 ± 8.3
	14 days	95.0 ± 4.5	5.0 ± 4.5	82.4 ± 5.1	17.6 ± 5.1
	28 days	86.7 ± 7.7	13.3 ± 7.7	85.5 ± 9.3	14.5 ± 9.3

Values represent the mean ± standard deviations. IC, Ischemic non-exercised control; IE, Ischemic exercise.

### Soleus cross-sectional area

The cross-sectional areas of the Type I and Type II fibers in the central portion of the soleus muscle were measured. Five days post-surgery, the cross-sectional area of Type II fibers on the paralyzed side was significantly larger in the IE group than in the IC group. Furthermore, comparing the cross-sectional areas of Type II fibers on the left and right sides on day 7 in the IC group, the cross-sectional area on the paralyzed side appeared reduced (Figure 2c), albeit not significantly. No specific or significant changes were observed in bilateral differences, comparisons between the IC and IE groups, or time-dependent comparisons based on postoperative days, despite minor fluctuations on both sides from 1 to 28 days post-surgery in Type I fibers (*p* = 0.69) and Type II fibers (*p* = 0.92) (Table 3).

**Table 3:** Fiber cross-sectional area of the soleus muscle in rats.

Group		Left soleus (unaffected side)		Right soleus (affected side)	
		Fiber CSA (μm^2^, *M* *±* *SD*)		Fiber CSA (μm^2^, *M* *±* *SD*)	
		Type 1	Type 2	Type 1	Type 2
IE	1 days	2,248.51 ± 308.37	1,679.47 ± 597.25	2,169.58 ± 115.44	1,571.27 ± 123.22
	3 days	1,899.41 ± 131.35	1,008.84 ± 162.82	1,948.91 ± 501.91	1,204.76 ± 147.08
	5 days	2,099.31 ± 158.55	1,415.18 ± 278.88	2,589.95 ± 74.01	1,947.06 ± 78.85
	7 days	2,205.29 ± 165.08	1,345.07 ± 139.14	2,350.42 ± 198.91	1,391.48 ± 51.82
	14 days	2,233.51 ± 202.84	1,758.32 ± 123.82	2,181.19 ± 359.66	1,825.64 ± 261.95
	28 days	3,013.14 ± 542.73	1,099.71 ± 897.73	2,665.24 ± 491.72	1,661.62 ± 811.17
IC	1 days	1,899.49 ± 187.59	1,460.34 ± 171.06	1,968.26 ± 246.52	1,631.98 ± 307.05
	3 days	2,065.41 ± 100.23	1,458.16 ± 69.68	2,051.63 ± 247.24	1,384.06 ± 171.35
	5 days	2,284.55 ± 170.84	1,315.61 ± 409.31	2,062.99 ± 296.07	1,233.06 ± 166.24
	7 days	2,243.19 ± 99.10	1,300.93 ± 102.34	2,287.45 ± 273.14	1,349.99 ± 285.01
	14days	2,157.73 ± 45.05	1,551.50 ± 46.13	2,228.90 ± 138.07	1,359.55 ± 194.07
	28days	3,028.35 ± 590.33	1,770.00 ± 1,176.21	2,725.59 ± 392.08	1,733.45 ± 755.05

Type 1 and Type 2 fiber cross-sectional areas (CSAs) of the soleus muscle in a rat stroke model.

There was no significant difference between Type 1 and Type 2 CSAs. Values are presented as the mean ± standard deviations (M ± SD). IC, Ischemic non-exercised control; IE, Ischemic exercise.

## Discussion

The present study investigated the effects of very early, low-intensity treadmill exercise initiated 24 h after focal cerebral infarction on neurological recovery, infarct evolution, and soleus muscle morphology in rats. Using a rat model, this study employed a simple research design to investigate ischemic stroke, the most prevalent type of stroke. We examined whether there were differences in the motor function (beam walking), neurological function, muscle fiber composition, and cross-sectional area of the soleus muscle between rats that performed a low-intensity treadmill exercise intervention starting immediately after cerebral infarction (24 h) and continuing for 28 days and those that did not. The principal finding is that early exercise significantly improved motor performance and neurological scores and suggested a reduction in lesion extent detectable by TTC staining, despite the absence of significant changes in soleus muscle weight, fiber-type distribution, or fiber cross-sectional area. This dissociation represents the central contribution of our study and provides important insight into the mechanisms underlying early functional recovery after stroke.

The present findings may reflect a temporal dissociation between central and peripheral adaptations following stroke. During the acute-to-subacute phase, exercise-induced recovery may depend predominantly on neuroprotection, lesion stabilization, and neural plasticity. In contrast, structural adaptations in skeletal muscle may require greater mechanical loading and emerge over a longer time course. Therefore, the absence of detectable morphological changes in the soleus muscle does not necessarily indicate an absence of biological adaptation, but rather suggests that central responses may predominate during early recovery. This interpretation is consistent with previous studies suggesting that neural adaptations may precede detectable skeletal muscle remodeling during post-stroke recovery (18, 20, 21).

The soleus muscle was selected for analysis because it is a key antigravity muscle with a high proportion of Type I fibers, making it particularly susceptible to disuse atrophy and neural impairment following stroke (30, 31). Immediately after cerebral infarction induction, both groups experienced a functional decline in the motor and neurological domains, along with weight loss (32). It also occurs in humans during the hyperacute phase (immediately after onset) (10, 33). Following a cerebral infarction, factors such as severe illness or prolonged bed rest can lead to sarcopenia (muscle loss) (comparison of changes in skeletal muscle mass after stroke categorized by the severity of motor dysfunction) (3, 11, 34) and alterations in skeletal muscle fiber composition (11, 12, 16, 35).

In contrast, in this study, although temporary weight loss and a corresponding decrease in soleus muscle mass occurred, no significant differences over time were observed in the composition ratio of soleus muscle fiber types or in the soleus muscle cross-sectional area. This indicated that the induced cerebral infarction originated in the middle cerebral artery territory, a common approach used in animal studies on cerebral infarction induction (22, 23, 31). This model does not induce severe physical symptoms (from an animal welfare perspective) and allows rats to perform essential daily activities, such as drinking and feeding, independently. Therefore, the animals in this study might also have functional impairments that allowed them to move freely within their cages and begin treadmill exercise 1 day after induction. The kinematic function observed in this assessment requires a high degree of physical balance (23, 25). Although the post-induction score was markedly low, no impairment hindered daily activities in this instance. Weight loss was observed due to temporary cerebral edema and other effects of cerebral infarction. However, by day 28 post-surgery, weight had increased significantly from pre-surgery levels, regardless of exercise status. Studies in humans have also reported that weight loss following cerebral infarction is significantly associated with reduced muscle mass and prognosis (36–38).

This suggests that whether a patient exhibits physical functional impairment at a level that prevents them from performing activities of daily living significantly influences weight control after cerebral infarction and is also a major factor in subsequent physical prognosis (39, 40). All animals were able to feed, drink, and move independently, and body weight recovered and exceeded preoperative levels by 28 days, suggesting preserved basic activity and nutritional status. We also examined the examined changes and differences following treadmill exercise-induced cerebral infarction. Previous studies have demonstrated that early rehabilitation intervention following cerebral infarction significantly influences subsequent functional prognosis (4–7, 23, 41). However, there is currently no consensus regarding the type and intensity of exercise interventions (10, 42). This treadmill exercise has been shown to be a low-intensity exercise in previous studies (43). One key feature of this study is its longitudinal design. The exercise protocol was deliberately designed as low intensity, prioritizing safety and feasibility in the early post-stroke phase rather than providing sufficient mechanical loading to induce muscle hypertrophy.

Because physiological indicators of exercise intensity, such as %VO₂max or metabolic equivalents, were not assessed, the term “low intensity” should be interpreted as an operational description based on treadmill speed, duration, and non-exhaustive conditions rather than a physiological classification. This limitation should be considered when interpreting the findings and comparing the present results with those of other exercise studies.

Rehabilitation interventions following human cerebral infarction vary in the type, frequency, and intensity of exercise, and this variability (40) contribute to the lack of consensus on the optimal approach for physical functional recovery (34). As demonstrated in the present study, early intervention with low-intensity exercise helps motor function and neurological recovery (6, 7, 23). However, exercise did not sufficiently alter the composition of the soleus skeletal muscle. Improved neurological scores and enhanced performance in the beam-walking task further indicate that exercise contributed to better motor coordination and balance, functions that rely heavily on central sensorimotor integration rather than muscle hypertrophy alone. These findings underscore the importance of neural recovery processes during the acute-to-subacute phase after stroke. Importantly, the absence of muscle structural change should not be interpreted as a failure of the intervention. Instead, it indicates that early low-intensity exercise does not exacerbate muscle deterioration, while still conferring central benefits. This finding is clinically relevant, as concerns regarding potential muscle stress or adverse effects often limit the implementation of early rehabilitation. Our results support the notion that safe, low-intensity mobilization can be initiated without detrimental effects on skeletal muscle integrity.

### Study limitations

Several limitations should be acknowledged. First, we were unable to examine signaling pathways or substances expressed in the skeletal muscle. Molecular markers of muscle atrophy or anabolic signaling were not assessed, and thus subcellular remodeling cannot be excluded. Structural preservation at the histological level does not preclude molecular adaptations that may precede morphological changes. It is important to investigate changes in skeletal muscle after the onset of cerebral infarction (44–48). However, the results obtained herein suggest that if animals can independently perform daily activities, including feeding and defecation, and regain body weight, disuse muscle atrophy and pathological changes in muscle structure are less likely to occur (42). This allowed for a 28-day longitudinal examination, which is difficult to perform in animal studies (44). While it is important to pursue a microscopic world to understand what is being expressed, it is fundamentally more important to prevent skeletal muscle changes after cerebral infarction and maintain function. Second, only the soleus muscle was examined. Although its antigravity function and high proportion of Type I fibers make it particularly relevant to postural control, responses in fast-twitch muscles may differ. Third, muscle function and neuromuscular activation were not directly measured, and cross-sectional area alone does not fully capture muscle performance.

Furthermore, early time points involved relatively small sample sizes, and TTC staining at chronic stages has inherent limitations in detecting tissue remodeling. Because variability estimates are less robust under such conditions, both positive and negative findings observed at the early time points should be interpreted as exploratory rather than definitive. These constraints were due to the longitudinal design and ethical considerations of animal experimentation and have been explicitly acknowledged to ensure transparent interpretation. This study employed a very simple design, and while the new findings are modest, its value lies in examining the subject at a macro scale rather than a micro scale under unified conditions that account for the background diversity often challenging in human research (12, 34, 39, 40, 49). Therefore, both positive and negative findings observed at the early time points should be interpreted as exploratory rather than definitive.

The findings of this study support the notion that if weight can be restored (36–38) through basic physical activities of daily living (such as eating and toileting) following cerebral infarction, negative changes in fiber composition (3, 32) and muscle fiber cross-sectional area of the soleus muscle, a skeletal muscle prone to atrophy after cerebral infarction (35), do not occur. This allows the skeletal muscle structure to be maintained. Following cerebral infarction, early low-intensity exercise intervention did not yield significant effects, such as muscle hypertrophy. However, in the long-term, exercise may further improve dexterous balance (beam-walking) and neurological function, potentially aiding in the recovery of damaged brain areas. Future research should examine this issue from a multifaceted perspective.

Despite these limitations, the present study provides important evidence that early, low-intensity exercise can facilitate neurological recovery and limit infarct progression without inducing muscle hypertrophy or preventing muscle deterioration simply because deterioration may not yet be present. These findings highlight the primacy of central mechanisms in early recovery and suggest that the goals of acute-phase rehabilitation should focus on neural protection and functional reactivation rather than muscle strengthening *per se*.

## Conclusions

This study used a very simple design to examine the soleus muscle, a skeletal muscle prone to atrophy and degeneration following cerebral infarction, over time, and to investigate whether early treadmill exercise intervention makes a difference. If animals independently performed daily activities, including feeding and elimination, and regained body weight, disuse muscle atrophy and changes in muscle structure were unlikely to occur, even without early rehabilitation intervention. However, while a low-intensity treadmill exercise intervention from an early stage did not yield effects such as muscle hypertrophy, it suggested the potential for long-term benefits: improvements in dexterous balance (beam walking) and neurological function, attenuation of lesion progression detectable by TTC staining, and possibly contributing to recovery-related neural processes. The findings suggest that consistent implementation of low-intensity rehabilitation interventions from an early stage over a certain period may serve as a catalyst for enhancing physical functional recovery.

## Data Availability

The raw data supporting the conclusions of this article will be made available by the authors, without undue reservation.
